# Clofazimine pharmacokinetics in novel rifampicin-resistant tuberculosis regimens: an analysis of the endTB and endTB-Q trials

**DOI:** 10.64898/2026.09.18.26363422

**Published:** 2026-09-20

**Authors:** Bernard Ngara, Belen P.Solans, Eunsol Yang, Pieter Van Brantegem, Lorenzo Guglielmetti, Francis Varaine, Maelenn Gouillou, Carole D. Mitnick, Allison N. LaHood, Michael L. Rich, Kwonjune J Seung, Lubbe Wiesner, Loren Hans, Rina Swart, Amanzhan Abubakirov, Kanat Khazhidinov, Anel Belgozhanova, Stephane Mpinda, Sesomo Mohale, David Holtzman, Dante Vargas Vasquez, Fanny Garcia Velarde, Sergio Mucching Toscano, Annum Aftab, Mahnoor S. Arshad, Azka Ashraf, Dinh Van Luong, Hanh T Nguyen, Ha TT. Phan, Sean Wasserman, Nelisiwe Ntuli, Rada Savic, Helen McIlleron, Gustavo E Velasquez

## Abstract

Introduction Rifampicin-resistant tuberculosis poses a significant threat worldwide. Clofazimine is considered important for the construction of effective individualized multidrug regimens to treat rifampicin-resistant tuberculosis. Our goal was to characterize clofazimine pharmacokinetics in novel combination treatment regimens and to identify participant characteristics associated with variations in drug exposure. Methods Clofazimine pharmacokinetic data were obtained from adults and adolescents enrolled in the PandrTB pharmacokinetic sub-study of the endTB and endTB-Q Phase 3 randomized controlled therapeutic trials for rifampicin-resistant tuberculosis. Plasma concentrations were analyzed using nonlinear mixed-effects modeling to quantify clofazimine exposure and explore the impact of relevant covariates. Results 100 participants from six countries with high burdens of rifampicin-resistant tuberculosis contributed pharmacokinetic data. Their median age was 34 years, 32% were female, 18% were living with diabetes mellitus, and 18% were living with HIV. Clofazimine plasma concentration values were best described by a 2-compartment pharmacokinetic model with first-order absorption. Co-administration with delamanid and HIV co-infection resulted in a 37% increase and 20% decrease in clofazimine bioavailability, respectively. Diabetes was associated with a 43% decrease in clofazimine clearance. Clofazimine remained in the body for a median of 2.5 years (95th percentile: 0.5 to 8) following 9 months of treatment. Conclusion Co-administration with delamanid, diabetes mellitus, and HIV were associated with variation in clofazimine exposure. We estimated that clofazimine remained present in the body for longer than two years in over half of participants following 9 months of treatment. Follow-up studies are recommended to confirm these associations before adjusting clofazimine dose or clinical care decisions.

## Full Text Availability

The license terms selected by the author(s) for this preprint version do not permit archiving in PMC. The full text is available from the preprint server.

